# Solution-focused approaches for treating self-injurious thoughts and behaviours: a scoping review

**DOI:** 10.1186/s12888-024-06101-7

**Published:** 2024-10-01

**Authors:** Lauren Jerome, Saba Masood, John Henden, Victoria Bird, Dennis Ougrin

**Affiliations:** 1 Youth Resilience Unit, Centre for Psychiatry and Mental Health, Wolfson Institute of Population Health, Queen Mary University of London, London, UK; 2 John Henden Consultancy Ltd, Taunton, UK; 3 Unit for Social and Community Psychiatry, Centre for Psychiatry and Mental Health, Wolfson Institute of Population Health, Queen Mary University of London, London, UK

**Keywords:** Solution-focused, Self-injurious thoughts and behaviours, Suicidal thoughts, Self-harm, Suicide, Scoping review

## Abstract

**Background:**

Self-injurious thoughts and behaviours are a major global public health concern, not least because they are one of suicide’s strongest predictors. Solution-focused approaches are a psychotherapeutic approach currently being used to treat individuals with self-injurious thoughts and behaviours but there is little published evidence of their use. We conducted a scoping review to provide a comprehensive overview of how solution-focused approaches are being used to treat self-injurious thoughts and behaviours.

**Methods:**

Publications describing a solution-focused approach being delivered to any individuals experiencing any form of self-injurious thought and/or behaviour were eligible for inclusion. Five databases were searched (EMBASE, PubMed, Web of Knowledge, PsycINFO, and Google Scholar) from inception to August 2024. Search terms contained keywords relating to both solution-focused and self-injurious thoughts and/or behaviours. Data were analysed using relevant steps from a narrative synthesis approach to summarise the participants, concepts, context and outcomes described in the included publications.

**Results:**

Twenty-four publications were included in the review. Publications demonstrated a global reach although the majority were published in the UK and USA. Five publications formally assessed and reported outcomes; two randomised controlled trials, one experimental pilot study, one case study, and one single group study. Only the Beck Depression Inventory was collected in more than one study (*n* = 4), with a range of other psychopathology and wellbeing-related measures. Three studies reported qualitative data, finding positive perceptions of the approaches by patients and clinicians. Fifty-one unique components were identified within solution-focused approaches. Often specific adaptations were described, or components were introduced, that specifically addressed suicide or self-harm. For example, identifying and working on goals related to reducing or stopping self-harm, or scaling questions that assess how suicidal someone currently feels on a 0 to 10 scale.

**Conclusions:**

This review demonstrates the application of solution-focused approaches for treating individuals with self-injurious thoughts and behaviours. The findings provide a comprehensive overview of how these approaches are delivered. The lack of outcome data and empirical studies highlights a need for more formalised evidence.

**Supplementary Information:**

The online version contains supplementary material available at 10.1186/s12888-024-06101-7.

## Background

Self-injurious thoughts and behaviours (SITBs) (including suicidal ideation, self-harm by self-injury or self-poisoning, and suicide attempts) are increasingly common [1] and are one of the leading causes of death globally, particularly in children and young people [2, 3]. They also strongly predict later completed suicide [4–6]. In 2022 5,642 individuals died by suicide in England and Wales [7], and approximately 700,000 people are estimated to die by self-harm globally every year [8]. This makes SITBs particularly important to treat effectively.

Self-injurious behaviours have many functions including, but not limited to, coping with emotions, to experience control, as a form of punishment, or to communicate distress [9]. The Interpersonal Theory of Suicide [10] proposes exposure to self-injurious behaviours reduces fear and pain, and increases relief, thus leading to capability for suicide. This theory also proposes that thwarted belongingness (a lack of reciprocated relationships) and perceived burdensomeness elicit suicide ideation, which can lead to an active desire and capability for suicide when combined with self-injurious behaviours. Premature removal of coping behaviours can lead to emotional vulnerability, so work around the behaviour to improve coping in a more healthy way is important. In order for this to be effective, it must fit with an individual’s theory of change and goals [11].

Various psychotherapeutic approaches are being applied to treating individuals with SITBs, with studies reporting mixed evidence and small effect sizes [12–18], leaving scope for improvement in how we approach treatment. Currently, the National Institute for Health and Care Excellence (NICE) guidelines only provide recommendations for the treatment of self-harm, which recommends Cognitive Behavioural Therapy (CBT) tailored to self-harm for adults, and Dialectical Behavioural Therapy for children and young people (DBT-A) [19]. However, the evidence for both of these treatments is of low quality and inconclusive [14, 15], and DBT-A still has very little evidence for self-harm in young people compared to NICE recommendations for other conditions [20]. Moreover, most interventions which show a reduction in suicidal behaviours have limited or no impact on suicidal thoughts [21]. Reducing suicide and self-harm is a national priority and focus on improving how we treat these is necessary [22].

Solution-focused approaches are another type of psychotherapeutic approach that has been applied to treating SITBs, but currently has little reported evidence compared to other approaches. These approaches are characterised by an orientation towards solutions using client strengths and resources to bring about desired change. Clear and concrete goals are utilised to facilitate the achievement of change [23]. These approaches were most famously conceptualised by de Shazer and colleagues in their development of Solution-Focused Brief Therapy (SFBT) in the 1980s [24], and popularised in the UK by the BRIEF centre [25]. These approaches were intended to be generic, suitable in many contexts, and practiced in a wide range of settings [26], but lack a strong evidence base.

Gingerich and Peterson [26] reviewed controlled outcome studies of SFBT finding it to be delivered in the broad areas of child academic and behaviour problems, adult mental health, marriage and family, occupational rehabilitation, health and aging, and crime and deliquency. They found 20 of 24 randomised studies reported a significant benefit from SFBT, with the strongest evidence being for adult mental health. They suggested there are six key characteristics of SFBT including; specific goals, the miracle question, scaling questions, searching for exceptions, compliments, and homework. Jerome, et al. [27] more recently conceptualised how solution-focused approaches are described in the adult mental health literature and found 16 components being delivered, including those identified by Gingerich and Peterson as well as additional components including utilising client strengths, collaborative working, an assumption of inevitable change, and considering the views of others. McKergow [28] notes there has been a shift in practice towards techniques used within solution-focused approaches being tools available to explore a client’s best hopes and enrich their description within a conversation, rather than a need to cover specific questions within a session.

Solution-focused approaches are well suited to treating SITBs because of their generic approach, focusing on whatever the client wishes to focus on, with the therapist leading from ‘one step behind’ rather than imposing a particular agenda on the discussions [27]. This supports sessions to be considerate of an individual’s perspective and goals. Being focused on their desired future and what it looks like when things go well, rather than unravelling a problem, is empowering and motivating, encouraging individuals to believe change is possible and within their capabilities. This is supported by feedback from clients who had undertaken a solution-focused approach in individual psychotherapy reporting liking how they were supported to recognise change is possible, their awareness of progress and things that are positive increased, their hope and motivation increased, and an absence of therapist evaluations or analyses of the past [29]. This is also supported by reports from individuals with lived experience of self-harm, who have undergone psychotherapy, that therapy was most beneficial when it was patient-led and focused on their goals which often went beyond the self-harm behaviour itself, rather than when an agenda is imposed by their therapist [30].

Despite reports of solution-focused approaches being applied to treating SITBs [31, 32], no review has summarised and described how these approaches are being used with individuals with SITBs. Although these approaches were designed to be generic and adaptable it is unclear whether any adaptations are made for working with individuals presenting with risk of suicide or self-harm. Characterising what is being delivered will help inform future interventions and practitioners who work with individuals with SITBs, and will add to the literature of therapeutic approaches delivered to individuals with SITBs.

Given the lack of studies investigating the effectiveness of solution-focused approaches with individuals with SITBs, we aimed to conduct a scoping review to provide an initial comprehensive overview of how these approaches have been applied to treating SITBs.

### Objectives

We aimed to identify the following specific objectives within this review;

- What specific populations have been targeted by these approaches? (i.e. ages, type of self-harm etc.)
- What are the main components of these approaches?
- What modes of delivery have been used?
- What is the intended treatment outcome of these approaches?

## Methods

This scoping review methodology was developed and conducted in accordance with the Joanna Briggs Institute (JBI) methodology for scoping reviews [33, 34], in conjunction with the Preferred Reporting Items for Systematic Reviews and Meta-Analysis Extension for Scoping Reviews (PRISMA-ScR) statement [35]. This review is registered at Open Science Framework: 10.17605/OSF.IO/YW5J2.

Scoping reviews typically define Population, Concept and Context (PCC) elements to guide their research question(s) and inclusion criteria. In our review, population was defined as participants either receiving, or intended to receive, the described approach, who were any age and experienced any form of SITB. Given the generic nature of solution-focused approaches, we chose to use a broad definition for our concept which would capture any publication self-described as ‘solution-focused’ as opposed to suggesting certain criteria must be present. This enabled us to characterise what solution-focused approaches are being delivered in the treatment of SITBs. We gave no restrictions on context. We used the PCC elements to define our inclusion criteria and data to extract and to organise our findings.

### Search methods

A full search strategy was developed based on search terms used in previous reviews of topics related to SITBs and solution-focused approaches. The following keywords were searched to characterise terms relating to:

i. solution-focused approaches (“solution*focus*” OR “solution*orient*” OR “solution*driven” OR “solution*based”) AND
ii. SITBs (suicid* OR “non*suicid*” OR NSSI OR Parasuicid* OR “Self*injur*” OR “self*cut*” OR “self*harm*” OR “Self*Mutilat*” OR Autoaggress* OR Automutilat* OR “Self*destruct*” OR “Self*immolat*” OR “Self*poison*” OR “Self*inflict*” OR “Kill ????self” OR “Kill ????selves” OR “ending own life” OR “taking own life” OR “thoughts of death” OR “fatal behavio? r*” OR “self*lacerat*” OR “overdos*” OR “self*defeat*” OR DSH)

No restrictions on dates were applied. Suggestions of relevant publications were obtained from key researchers and experts in the field, and forward citation tracking and reference list screening were performed for each included publication to identify any further relevant publications.

EMBASE, PubMed, Web of Knowledge, and PsycINFO were searched from inception to February 2023. An updated search was conducted in August 2024. Google Scholar was also searched using the same search terms primarily to identify any relevant publications that were missed by the other databases.

### Study selection

Following the search, all identified citations were collated and uploaded into Endnote (version X9), and duplicates were removed. The remaining citations were transferred to Rayyan for screening.

First, titles and abstracts were screened against the eligibility criteria, followed by a full-text screening of any remaining publications. 50% of the publications were screened by the first author (LJ) and 50% by the second author (SM), with 10% independently assessed by both authors to check for agreement at each stage. Any disagreements were resolved through discussion, with only ∼ 3% disagreement at each stage. Reasons for exclusion were recorded at the full-text screening stage.

The inclusion criteria encompassed studies of any study design, book chapters, protocols and some grey literature (including conference abstracts, theses, preprints, guidelines, policy documents, or any other form of report) where the approach described meets all other criteria and describes an approach actually delivered or intended for delivery, to individuals with SITBs. Publications identified in the search could be in any language but were only kept for inclusion if an English translation was available. We included publications where the participants, or intended recipients, of the described approach were individuals of any age experiencing any form of SITB. The publication had to self-describe their approach as solution-focused or being based on a solution-focused model and described in sufficient detail to extract information on its content.

### Extraction

Data extraction was performed by both LJ and SM, with both authors extracting data for 50% of the included publications. Twenty-five-percent of the publications were independently extracted by both authors to check for agreement in the information extracted and to pilot the extraction spreadsheet, which was reviewed after independent extraction from three publications. As a result, the extraction spreadsheet was updated adding clarity to the description of the data to be extracted in each column, and two columns were removed where the information was already captured elsewhere.

Data were extracted that contained details of the publications, including their year of publication, country, study design, type of publication, and any limitations. Participant data were extracted, including eligibility criteria, the definition of SITB given, and intervention and control group descriptions (if relevant). Information on the described approach was extracted, which included the main components of the approach as described in the publication, the mode of delivery, what the approach is looking to change or address, and the length of treatment. Finally, for publications reporting outcome data, details of any outcomes and results were extracted if possible. No missing or additional data were identified requiring contact with the relevant author.

A critical appraisal of the included publications was not conducted for this review. Critical appraisals are not typically included in scoping reviews, since they aim to provide an overview of a body of literature regardless of its quality, as opposed to only selecting high-quality evidence to answer a particular question [33, 34]. Since this review aims to provide an overview of approaches described as solution-focused being applied to treating SITBs, a formal assessment of quality was not deemed relevant to the aims.

### Analysis

Data were analysed using relevant steps from a narrative synthesis approach based on guidance by Popay, et al. [36]. Given the aim of this scoping review was to provide a descriptive overview of how solution-focused approaches are used to treat SITBs, only some steps of the narrative synthesis approach were relevant given meta-analysis or theory development were not within our aims. Steps were chosen that allowed us to synthesise findings from across our included publications, explore relationships between different characteristics, and reflect critically on our synthesis process. Data relating to participants, context, and outcomes were primarily synthesised using tabulation, groupings and graphs to explore and present the relevant findings.

To synthesise the findings relating to concept, textual descriptions from the extracted data underwent a basic content analysis [37] to categorise the key components that were explicitly described by the author(s) in the publications. First, an initial deductive approach was taken, given the existing literature on solution-focused approaches and the recent conceptual review conducted by LJ [27]. A data dictionary was prepared describing the content to be coded, i.e., the type of question or activity described. After familiarising with the data, each extract was coded according to the data dictionary. Where content did not fit into an existing code category, an inductive approach was taken, coding the content into a new category. Once all data had been coded, the categories were reviewed and changed or assimilated where it made sense to do so and aided with understanding the data. SM independently coded 12.5% of the included extracts to check for any differences in how content should be coded using the same data dictionary. The names of the approaches described and what they were looking to change or address were also tabulated and grouped to provide an overview.

Finally, we reflected critically on our synthesis as the review progressed through discussion and taking note of anything which may have impacted our approach and findings. Given LJ’s background in researching solution-focused approaches, we were aware her existing knowledge of the field may lead to biases in understanding the approaches in our included publications. Discussion of the findings with SM, who does not have the same experience, the wider research unit LJ sits in, and experts in the field, including clinical practitioners working with solution-focused approaches and individuals with SITBs, helped to consider other perspectives in our synthesis.

## Results

**Fig. 1:**
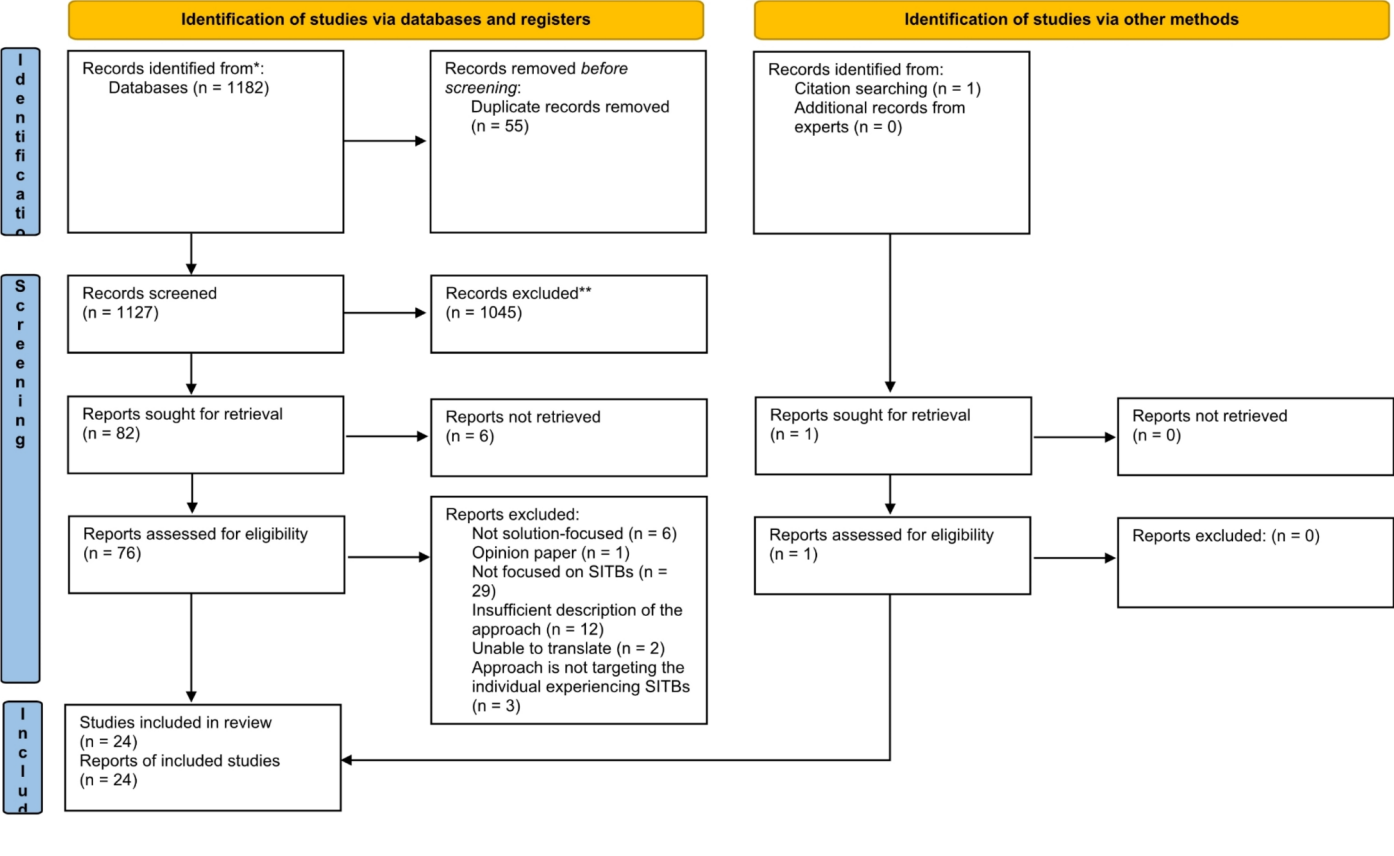
PRISMA-ScR flow diagram [38]

The search found 1183 records in total, with 55 removed as duplicates. One-thousand-forty-five were excluded at the title and abstract screening stage. Seventy-seven publications in total had their full text screened, and 24 ultimately were included in the review (see Fig. 1). Included publications were published between 1998 and 2023, in Australia (*n* = 3), Canada (*n* = 1), Finland (*n* = 1), India (*n* = 1), Japan (*n* = 1), the Netherlands (*n* = 1), Turkey (*n* = 1), the UK (*n* = 6) and the USA (*n* = 9). The types of publications included encompassed empirical research (*n* = 10), conference notes (*n* = 2), a literature review (*n* = 1), a thesis (*n* = 1), and book chapters outlining evidence, guidelines and techniques for a variety of practitioners (*n* = 10). A summary of included publications is provided in Table 1.

**Table 1:** Summary of included publications

Lead author	Year of Publication	Country	Participants/ Population of Interest	SITB Definition	Setting and Delivery	Study Design/Type	Name of Approach	Summary
Ayar [39]	2021	Turkey	Sixty-two adult inpatients with depression, with a moderate level of suicide risk	Suicide probability risk is measured using the Suicide Probability Scale (SPS)	Individual sessions delivered by a researcher, in-person, in an inpatient unit of a psychiatric hospital	Research paper – Randomised controlled trial	Solution-Oriented Approach Intervention	Investigated the effect of their intervention, delivered over 6 sessions, on depressive symptoms, social functioning, and suicide risk. The intervention group had improved social functioning and lower suicide risk compared to pre-intervention and a control group receiving routine clinical care.
Baijesh [40]	2018	India	Students	Suicidal ideation	Clinical Psychologist providing in-person individual sessions in a University Counselling Centre	Research paper – Case study	Solution-Focused Brief Therapy	SFBT techniques were used with a student client presenting with suicidal ideation. Scores on outcome measures at baseline, post-intervention and at follow-up are provided.
Bannink [41]	2015	USA	Suicidal persons’	Suicidal/in crisis	Therapist delivering individual sessions for crisis intervention	Book chapter – designed to equip clinicians with ready to use tools for working with clients	Solution-Focused Brief Therapy	Describes how solution-focused questions can be used with clients in crisis, in particular, applied to those who are suicidal.
Bliokas [42]	2019	Australia	Suicidal adults presenting to an Emergency Department	Suicide attempt or deemed at high risk for suicide	Individual sessions delivered by peer workers and mental health clinicians, either face-to-face or over the telephone, beginning from their presentation in an emergency department to outpatient	Research paper – Protocol paper	Aftercare Intervention	Investigating the effect of an aftercare suicide prevention intervention for individuals who present to an emergency department following a suicide attempt or with significant suicide risk. The intervention targets suicidal thoughts and behaviours and primarily aims to reduce re-presentations to ED, through a mix of sessions with a mental health clinician and peer workers.
Buchholz-Holland [43]	2017	USA	Students	Suicidal ideation	School social worker providing counselling in-person, in school, in individual sessions	Book chapter – to provide school based mental health practitioners with steps to respond to students with suicide ideation	Solution-Focused Approach	Outlines the prevalence of student mental health issues and the risk and protective factors. Then describes how solution-focused approaches differ to other approaches such as CBT, and applies the techniques to students struggling with suicidal ideation.
Cole-King [44]	2018	UK	Suicidal persons’	Not specified	Primary care practitioners delivering individual sessions either face-to-face or over the telephone	Book chapter – offering evidence and practical strategies for primary care and general practitioners	Solution-Focused Therapy	Gives an overview of what is known about risk factors for self-harm and suicide, and the need for assessment and awareness particularly focusing on clinicians in primary care. Discusses the Bank of Hope set of coping strategies and effective safety plans, before going on to discuss how SFBT can be applied to individuals at risk of suicide.
Finlayson [45]	2023	USA	Suicidal persons’	Suicidal ideation	Therapist providing telehealth services to individuals with the option to involve their family	Research paper – Description of an approach	Solution Focused Telemental Health Crisis Intervention	Describes telemental health, its application in suicide intervention, the applicability of SFBT to telemental health and specifically how it can be used to address clients presenting with suicidal thoughts in telehealth sessions.
Fiske [46]	1998	Netherlands	Suicidal persons’	Not specified	Therapist providing clinical suicide prevention work in individual sessions	Book chapter – describes therapeutic techniques which may be useful in clinical suicide prevention work	Solution-Focused Brief Therapy	Provides an overview of the philosophy and techniques of SFBT, in the context of therapy with suicidal persons, and then directly links SFBT techniques to Shneidman’s commonalities of suicide and discusses how SFBT can address the needs and challenges of these.
Fiske [47]	2008	USA	Suicidal persons’	Suicidal thoughts or behaviours	Therapist delivering in-person or over the telephone individual sessions	Book chapter – offers practice principles and practical tools to therapists	Solution-Focused Therapy	Provides a description of the SFBT approach and how it is relevant for individuals struggling with suicide. Specific techniques and how they can be applied to working with this group are described and explored, and examples of each technique are given.
Fiske [48]	2017	USA	Suicidal persons’	Not specified	Individual sessions provided for suicide prevention	Conference notes	Solution-Focused Brief Therapy	Outlines 20 reasons to use SFBT for suicide prevention.
Fiske [49]	2017	Japan	Suicidal persons’	Not specified	Individual sessions provided for suicide prevention	Conference notes	Solution-Focused Brief Therapy	An overview of SFBT for suicide prevention.
Fiske [50]	2018	UK	Individuals who have experienced trauma	Not specified	Group or individual in-person sessions	Book chapter – clinicians illustrate how to apply SFBT to traumatic experiences and clinical cases	Solution-Focused Brief Therapy	Provides a summary of how SFBT could be used to prevent suicide in the aftermath of trauma.
Guterman [51]	2013	USA	Suicidal persons’	Not specified	Counsellor providing individual sessions	Book chapter – clinical techniques and case studies illustrate how the model can be used	Solution-Focused Counselling	Describes a solution-focused approach and particular techniques for assessing and intervening with suicidal clients. The approach’s relation and applicability to hope and theories of hope and suicide are given with case examples.
Henden [52]	2017	UK	Suicidal persons’	Suicidal ideation	Therapist/ counsellor/ practitioner providing in-person individual sessions	Book chapter – practical guidance to individual suicide prevention	Solution-Focused Approach	Provides an overview of the philosophy and techniques of SFBT, in the context of therapy with suicidal persons, providing specific ways SFBT techniques can be adapted for working with this patient group. Explores the mechanism by which the approach can work with this group.
Kondrat [32]	2010	UK	Suicidal persons’ presenting to an Emergency Department	Suicidal ideation	Individual, in-person sessions	Research paper – Literature review	Solution-Focused Brief Therapy	Summarises how SFBT can be used in an emergency room setting.
Laydon [53]	2008	UK	Suicidal persons presenting in Emergency Departments	Deliberate self-harm	Liaison Psychiatry Team (including nurses, medical and social work staff) delivering individual, in-person sessions in the Emergency Department with follow-ups in clinic	Research paper – Describing their approach and historical findings	Single-Session Solution-Focused Brief Therapy	Describes developments in the use of a solution-focused approach with individuals presenting to A&E following self-harm, how this has been incorporated into initial assessments as well as subsequently introducing follow-up sessions and a feedback letter. Findings demonstrate the solution-focused approach to the initial assessment showed some effectiveness.
Lefranҫois-Crotty [54]	2013	Canada	Adolescents	Self-harm	In-person, group sessions delivered in residential facilities	Thesis	Solution-Focused Approach to Art Therapy	The value of art therapy with adolescent girls who struggle with self-harming behaviours is addressed through a contemporary lens. The suggested interventions were created within the frame of a solution-focused approach.
McAllister [55]	2009	Australia	Suicidal persons presenting to an Emergency Department	Self-harm	Nurses delivering in-person sessions in the Emergency Department and acute care areas	Research paper – A qualitative study	Solution-Focused Nursing	Evaluates the effectiveness of a solution-focused education intervention in extending and improving emergency nursing responses to patients who present because of self-injury. Qualitative results from interviews conducted with nurses who undertook the training in the approach are described.
McAllister [56]	2008	Australia	Suicidal persons presenting to an Emergency Department	Self-harm	Nurses delivering in-person sessions in the Emergency Department and acute care areas	Research paper – Cross-sectional survey	Solution-Focused Nursing	Evaluates the effectiveness of a solution-focused education intervention in extending and improving emergency nursing responses to patients who present because of self-injury. Quantitative results are provided from a survey of nurses who undertook training in the approach, assessing their perceptions of nursing and professional self-concept.
Rhee [31]	2005	USA	Callers to a suicide hotline	Not specified	Therapists providing individual telehealth sessions	Research paper – Randomised controlled trial	Solution-Focused Brief Therapy	Examined the efficacy of Common Factors Therapy and SFBT compared to a waitlist control, conducted exclusively over the phone. Both treatment conditions improved compared to the waitlist controls.
Selekman [57]	2006	USA	Adolescents who self-harm	Self-harm	School social workers and mental health counsellors, delivering sessions in-person, in school, predominantly individual sessions with some group activities	Book chapter – discusses an integrative approach to stopping self-harm by a psychotherapist	Integrative Solution-Oriented Brief Therapy Approach	Describes solution-oriented techniques that can be used with adolescents who self-harm.
Selekman [58]	2008	USA	Adolescents	Self-harm	School social workers and mental health counsellors, providing in-person, individual sessions in schools	Book chapter - discusses an integrative approach to stopping self-harm by a psychotherapist	Solution-Oriented Therapeutic Approach	Gives background to self-harming adolescents in the school environment and then provides several techniques from a solution-oriented therapeutic approach to be used with self-harming adolescents.
Tapolaa [59]	2010	Finland	Adults presenting to an Emergency Department with self-harm	Deliberate self-harm	Individual, in-person sessions were given by trained advanced level Psychology students in the Emergency Department	Research paper – Randomised controlled pilot study	Acceptance and Commitment Therapy with Solution-Focused Brief Therapy	Explored the effectiveness of a four-session intervention combining elements of Acceptance and Commitment Therapy and SFBT to prevent deliberate self-harm, compared to a treatment as usual control group. Both groups improved on various outcome measurements, with the intervention group showing more improvement at a 4-month follow-up.
Wiseman [60]	2003	UK	Suicidal persons presenting in Emergency Departments	Deliberate self-harm	Liaison Psychiatry Team (including nurses, medical and social work staff) delivering individual, in-person sessions in the Emergency Department	Research paper – Single group study	Solution-Focused Brief Therapy Risk Assessment	Describes the risk assessment model used for patients presenting with deliberate self-harm to A&E in the Tees and North East Yorkshire NHS Trust and how this was adapted to incorporate elements of SFBT. A small study looking at its impact is described which found only one participant (out of 40) re-presented during the study period.

### Participants

Recipients of the approaches were most often described as suicidal (*n* = 9), with it being unclear whether this referred solely to suicidal thoughts or also behaviours. Eight approaches focused on self-harm, five on suicidal ideation, and two on individuals who had made suicide attempts. In many cases, it was also unclear what age group were the intended recipients; six publications described the approach as being delivered to individuals attending an emergency department, nine to suicidal persons, and one of each to callers to a hotline and individuals who had experienced trauma. Five approaches focused on adolescents/young adults, and two on adults (one on adult inpatients specifically).

Given the lack of specificity in most approaches regarding their intended recipients, beyond them being suicidal, for the remainder of the results the term suicidal is used to refer to individuals where there was no further specificity of their SITB presentation.

### Concept

Approaches were most commonly named Solution-Focused Brief Therapy, with other names being given that pertained to their specific approach (see Table 1 for the name given to the approach in each publication).

Coding of the key components that were described in the publications identified 51 unique components. Additional File 1 gives a full list of the components including which publications they appear in and examples. Perhaps unsurprisingly, identifying and working on goals was the most commonly described component, followed by rating and scaling questions, and identifying strengths, skills, and resources. No component was described in every included publication.

Twenty-eight components made specific reference to SITBs in some, not all, of the descriptions. Most often this was by specifying within questions that SITBs were the issue that needs to be addressed or thing to change:

‘On a scale of 1–10 (where 1 stands for very suicidal and 10 stands for not at all), how suicidal do you feel right now? What would you be doing/thinking about/feeling to be another half-point higher?’ [52, p. 132] Rating and Scaling.

Sometimes components were described as being introduced to directly tackle SITBs, such as education on the mechanism of self-injury and alternative coping skills:

‘Skill deficits and excesses, such as the mechanism of self-injury which can be very effective in managing pent-up distress, are addressed. Together, the nurse and client might work on building a repertoire of coping skills’ [55, p.2840] Education.

Or components were introduced that were to be used specifically when an individual is in crisis, such as safety plans or resource forms to be used by the client and clinician:

‘A one-page document that identifies an emergency contact person, the nearest hospital, the local emergency number, and re-states the client’s address for ease of access. The use of a resource form gives clinicians a document that can be used in moments of crisis’ [45, p.55] Resource form.

It is important to note that many descriptions of these 28 components remained generic and made no reference to SITBs. Additionally, 23 of the components did not make any specific reference to SITBs in any of the publications, instead describing components that focused on whatever the client deemed important:

‘At the beginning of second and later sessions, positive change can be elicited by asking: - What has been better since we last met?’ [46, p.189] Identify and maintain change.

Or general solution-focused techniques important for the clinician to demonstrate:

‘should use the client’s strengths, keywords, beliefs, and metaphors connected to their major skill areas as much as possible to help foster a cooperative relationship with them’ [58, p.112] Develop a working relationship with the client.

### Differences based on SITB or population group

We also explored whether the components described varied depending on which population or SITB the approach was focusing on.

Homework was more likely to be described in approaches targeting young people and self-harm. Education and activities within sessions were only described for approaches addressing self-harm. Creating actions and reminders were only described in approaches focused on individuals who had made suicide attempts. Exploring pre-session change was most often described in approaches addressing suicidal ideation. Other components were present in a mix of approaches.

### Context

**Fig. 2:**
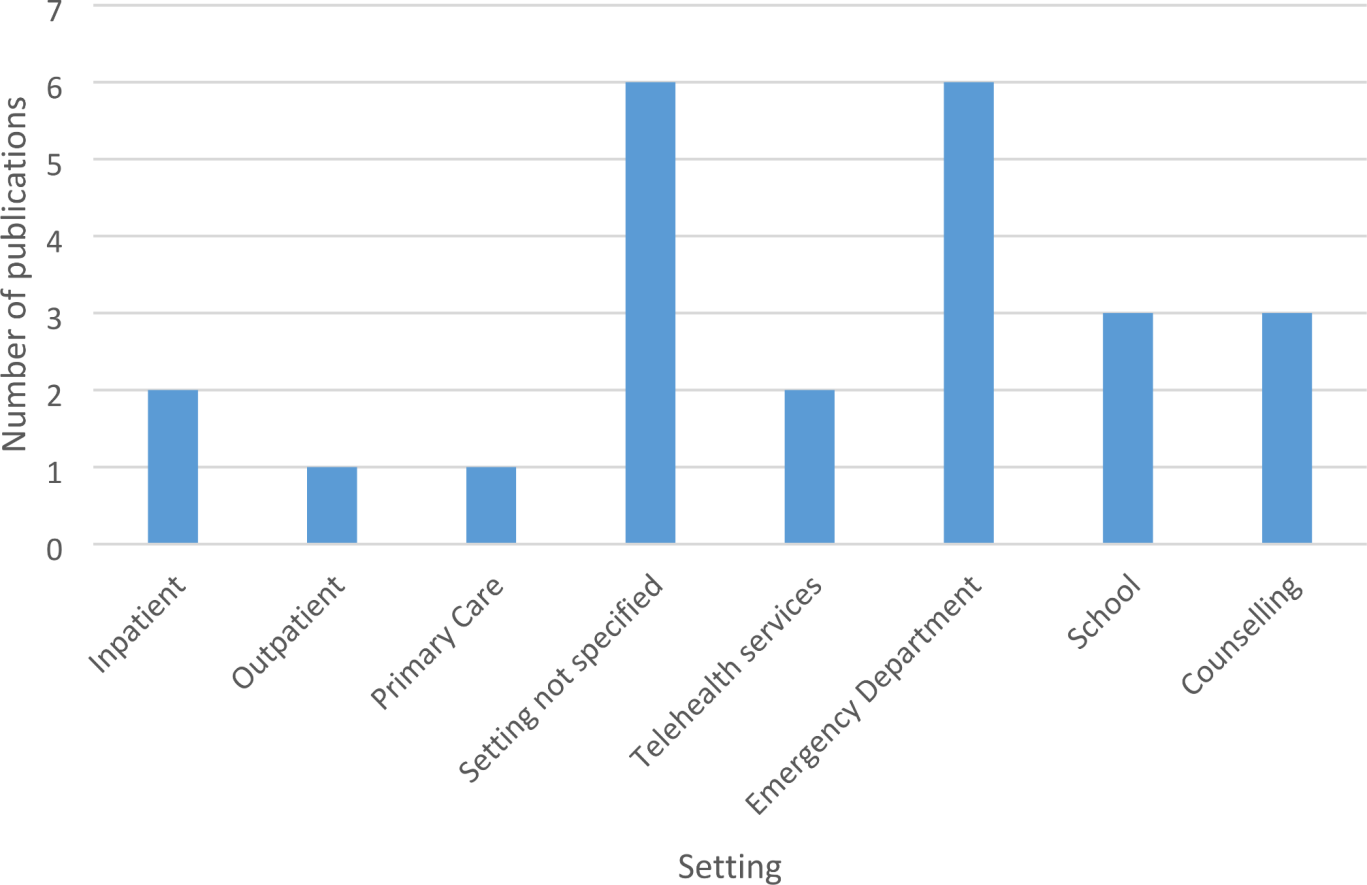
Delivery setting

Figure 2 displays the settings the approaches were delivered in.

Setting not specified is where the approach was being delivered in a therapeutic setting providing suicide intervention/prevention, but not specifically defined in the publication.

The majority of the described approaches were delivered in-person (*n* = 14). The remainder were either delivered over the telephone (*n* = 2), could be either in-person or over the phone (*n* = 3), or it was unclear in the publication if there was an intended mode of delivery (*n* = 5).

Additionally, the majority of the approaches were delivered to individuals (*n* = 20), with just one publication each being delivered; (i) in a group, (ii) delivered either as a group or to an individual, (iii) to an individual with some family involvement, or (iv) primarily to an individual with some group activities.

The number of sessions reported ranged from one to 21, although the actual number of sessions being delivered in practice was most frequently not reported or was described as ‘however many sessions are required’ [47, p.71]. The length of each session ranged from 30 to 60 min, and the total length of treatment ranged from one-session to 12-weeks. However, most often these details were not specified within the texts.

### Outcomes

Most publications aimed to reduce the repetition of self-harm or risk of suicide, with others looking to improve various mood and mental health-related outcomes, or a mixture of these. Some publications described seeking to achieve outcomes that the recipient decides on, even if they have nothing to do with their self-harm behaviour.

Five publications formally assessed quantitative participant outcomes that consisted of two randomised controlled trials, one experimental pilot study, one case study, and one single group study. The single-group study conducted by Wiseman [60] only reported repetition of self-harm at follow-up, where just one of 40 participants reported having repeated self-harm during the study.

In the other four publications a variety of outcome measures were collected. Only the Beck Depression Inventory (BDI) [61] was used in all four publications, with every other measure used uniquely. These included measures of general psychopathology, anxiety, quality of life, emotion regulation, hopelessness, and measures of suicide probability, suicidal ideation and self-harm. Additionally, two studies reported survey data on participants’ and clinicians’ perceptions of the approach.

Qualitative data were reported in three studies [40, 53, 55] although only one had a qualitative study design [55]. All three reported positive perceptions of their intervention from both participants and clinicians, either through anecdotal reports [40, 53] or from interviews conducted with nurses 2-weeks after they had received training in their intervention [55].

Publications not reporting outcomes included; a literature review, a protocol paper, conference notes, an intervention description, and several book chapters. Book chapters and conference notes primarily sought to describe practical techniques and steps various practitioners can take when working with individuals with SITBs. Further detail on each publication is included in Table 1.

A full list of publications reporting outcomes and their raw scores, as well as the themes identified by McAllister, et al. [55] are included in Additional File 2.

## Discussion

This scoping review provides a comprehensive overview of the use of solution-focused approaches for treating SITBs. Overall, the approaches have been used with individuals with a range of presentations and in a range of settings, most often being intended to be delivered in-person in individual sessions. We identified 51 key components being described in our included publications, some of which were tailored to specifically address SITBs. Only five studies measured quantitative outcomes and three reported qualitative data. This highlights a need for more high quality research studies to investigate these approaches in order to assess their impact and effectiveness. Indeed, research into treatment for SITBs lacks high quality conclusive evidence in general [14, 15]. Recent and ongoing large randomised studies are seeking to investigate the effect of interventions based on Psychodynamic therapy and Cognitive therapies for individuals who self-harm [62–64]. Exploration of solution-focused approaches in similar research programmes would benefit our knowledge of how to effectively support individuals with SITBs, and are beginning to be investigated in studies like the ASsuRED study, which includes solution-focused follow-up sessions following presentation to an emergency department having self-harmed [65].

The 51 key components we identified described in these approaches generally mirror those commonly described in solution-focused approaches used in other populations. They encompass those identified by both Jerome, et al. [27] and Gingerich and Peterson [26] in earlier reviews. This demonstrates consistency of this review’s included approaches with other applications of solution-focused approaches, and the endurance of what a solution-focused approach is understood to be. However, no component was described in every included publication. This could be due to the general perception of solution-focused approaches as generic and adaptable, so the use of particular components can be tailored to fit individual needs and contexts, which we found some evidence of when exploring our findings. Moreover, McKergow’s [28] observation of a shift in practice towards techniques being available tools to use within a solution-focused conversation, may explain why components are not consistently described. Solution-focused approaches have a range of techniques to draw upon, depending what the situation requires.

The components we found with adaptations specific to SITBs were most often techniques that would typically be tailored to whatever the client chooses to discuss in the session, for example the miracle question where the future scenario is whatever the client hopes to achieve. Additionally, SITB specific components were described that to our knowledge are not typically present in other applications of solution-focused approaches, such as educational materials on self-harm and coping strategies [55]. For solution-focused approaches to be generic and adaptable, generally it is considered inappropriate for the clinician to direct the conversation towards a particular agenda. Instead, the client is viewed as the expert leading the conversation, with the clinician taking a not knowing stance [27]. What was unique in our findings was the clinician seeming to dictate the focus of the discussion towards the outcome being alleviation of SITBs. These subtle variations of the approach to address the specific needs of this group may reflect the need for professionals to impose an agenda when there is a greater concern for an individual’s safety that needs addressing. However, it is important to note not all descriptions of components had adaptations, and many components had no adaptations mentioned at all. This suggests some discord in whether imposing an agenda that addresses SITBs directly is appropriate or needed in this context.

Given the reports of individuals having undergone psychotherapy following self-harm reviewed by Haw, et al. [30] suggest that an agenda imposed by therapists felt disempowering, invalidating, and created a power inequality within the therapeutic relationship, it seems counterintuitive for clinicians to dictate the focus of the questions to be concerning SITBs. Instead remaining generic and open to whatever the client chooses to focus on may be preferable and more conducive to a positive therapeutic relationship when working with individuals with SITBs. Additionally, the evidence currently is uncertain as to whether interventions which provide information and support, such as education on coping skills, are effective at reducing self-harm [66]. With this in mind, it appears crucial to test these solution-focused approaches being delivered to individuals with SITBs to better capture which components are indeed effective in treatment.

Solution-focused approach’s generic and adaptable nature may also contribute to the wide variety of settings, geographical locations and modes of delivery that we found in our included publications. Being generic and adaptable would lead to their ability to be incorporated into different health services, in health care systems in different countries that may vary greatly. Although most approaches were described as being delivered in-person and in individual sessions, we found some examples explicitly being delivered in other formats. That Rhee, et al. [31] found positive outcomes after delivering their approach over the telephone also provides some initial support for the ability of the approach to be delivered flexibly. Although we found the included approaches were described for delivery in a range of settings, there was little evidence of their effectiveness reported. Exploration of outcomes in approaches delivered across different settings would provide evidence to support their use.

Emergency departments were the most commonly specified setting the included approaches were delivered in. This could be explained by emergency department settings being where individuals with SITBs tend to present. However, Marchant, et al. [67] found that primary care settings actually see the highest incidence of presentations following self-harm, yet we only found one publication targeting primary care settings. It could also be that the brevity with which solution-focused approaches were originally conceptualised as being delivered in is particularly suitable for emergency department settings. Or the focus as an approach on positive and presuppositional language (utterances which assume something to be true i.e. when you are better what will you be doing), which conveys hope and optimism from the beginning, may be especially important with individuals in crisis. The finding by Wiseman [60] that just one out of 40 participants repeated self-harm during their study, after receiving a solution-focused approach in an emergency department setting, provides some preliminary evidence this is an appropriate place for these approaches. It may also be that solution-focused approaches might be suitable to only a subset of clients presenting at emergency departments [68], although clinicians appear to favour these approaches in emergency situations [69]. Further investigation of the use of the approach in different, and particularly primary care, settings would be beneficial.

Our results demonstrated a lack of consensus in the number of sessions delivered and the length of the sessions themselves, as well as the overall length of treatment delivered to a client. This could be due to the nature of the originally conceptualised solution-focused therapy approach, where it was intended to be generic enough to fit with any model of care, and any number of sessions the client deemed sufficient. As most publications did not report a number of sessions or length of treatment our ability to draw any inferences about whether the number of sessions related to particular components or characteristics of the approaches is limited. However, the ambiguity surrounding the length of treatment could also be due to their following the approach’s original conceptualisation, and remaining open to however many sessions the client believes are necessary.

Education strategies and activities within sessions were only described in included publications that were focused on self-harm. These are also components which do not seem to be present in other applications of solution-focused approaches. Many treatment approaches for SITBs focus on skills development [70], and the NICE guidelines recommend identifying individualised coping strategies for individuals who have self-harmed [19]. The inclusion of education and activities to improve coping in our publications may reflect an attempt to incorporate learning of alternative coping skills, which may be a particularly important focus for individuals who have self-harmed. However, as noted previously, it is unclear whether education on self-injury and coping does lead to positive outcomes [66].

Despite the use of risk assessments being widespread in mental health services, particularly for individuals presenting with SITBs [71], they were rarely mentioned in our included publications. This may be because they are seen as a separate part of practice to the solution-focused approach, and thus not described within our included approaches. NICE guidelines for self-harm advise against the use of risk assessments for risk prediction or treatment allocation, instead focusing more on safety and coping strategies [19]. The solution-focused approaches we identified fit well with these guidelines. The adaptations to typical solution-focused techniques we found in the included publications mainly explored what feeling safer, or an absence of SITBs, would look like, and additional techniques focused on self-harm included education on coping. The approach in this context therefore is primarily focusing on risk reduction and becoming safer, as opposed to focusing on explicit, direct risk assessment.

Whilst we highlighted and summarised the outcomes measured in our included publications, it was inappropriate to make any conclusions about effectiveness given the lack of quality assessment in this review. Moreover, it was not the aim of the scoping review to make such conclusions. Of the 24 publications included only five formally assessed outcomes, which included a range of measures with only the BDI being collected in more than one study. We also found a range of study designs, with only two randomised controlled trials and one formal qualitative study design. There are many practice-based reports of the success of solution-focused approaches in treating individuals with SITBs which have not been formally demonstrated in the literature [52], leaving solution-focused approaches as lacking published evidence. More research into solution-focused approaches used to treat SITBs using rigorous research designs could seek to measure similar outcomes as those in our five publications. This would enable the synthesis of findings to make better conclusions about solution-focused approach’s effectiveness for treating SITBs. Moreover, formalising evidence occurring in practice through both quantitative and qualitative research would benefit the field by providing more evidence of their effectiveness and appropriateness.

Although most publications described seeking to improve outcomes related to self-harm, suicidal thoughts, or other mental health-related outcomes, some described their targeted outcomes as that which the client decides on, even if it seemingly has nothing to do with their self-harm behaviour [57]. This again seems to relate to this idea of solution-focused approaches being generic, adaptable, and without any agenda on the part of the clinician – instead being totally centred on the client’s wishes. It would be interesting in future research to include investigation of these approaches’ effectiveness with the client’s chosen goal(s) as outcomes, as opposed to pre-determined measures of psychopathology, for example.

### Strengths and limitations

A strength of this review is the use of a systematic search. This ensured we captured a wide range of sources of evidence to understand how solution-focused approaches have been used with individuals experiencing SITBs, including publications that may describe a totally unique approach. This also ensured a thorough search of the literature, with a large number of publications screened for inclusion, enabling us to provide such a comprehensive overview. Second, the review team consisted of a mix of researchers familiar with solution-focused approaches or not, and the findings were discussed with both experts in the field and other mental health researchers familiar with different approaches. This ensured a critical reflection on the findings, and that they were not only interpreted in line with the researchers’ existing understandings of the approach in question. Third, this is the first review of its kind, providing an initial overview of solution-focused approaches use in treating SITBs, which will support further research exploring their use in treating SITBs.

A limitation of this review is that most included publications used the term suicidal generically and without specification of whether this applied to suicidal thoughts or also behaviours. This limited our findings as it is unclear whether there may be more substantial differences in how these approaches are used depending on specific types of SITB. Our findings do indicate there may be some specific techniques that are used with, for example, individuals who present with self-harm as opposed to suicidal thoughts. Moreover, different forms of self-harm (i.e. non-suicidal self-injury, suicide attempt) and self-injurious thoughts are recognised as distinct clinical syndromes [72]. Although making distinctions between suicidal and non-suicidal behaviour is an ongoing debate [73], there does appear to be a distinction between thoughts and behaviours, with treatment and outcomes often focusing on behaviours [21]. There may be a need to recognise differences between thoughts and behaviours when approaching treatment rather than a generic approach to treating individuals who are ‘suicidal’ whether that be due to thoughts, behaviours, or both. Second, often the included publications gave vague definitions of the specific characteristics of the approach and its delivery, making it difficult to discern specifics such as the setting or format the approach was delivered in. Again, this limits any comparisons we can make based on these characteristics. Moreover, we were limited in our synthesis to what the author(s) of the included publications explicitly described in their approaches. There may be other components or modes of delivery intended that we were unable to capture as a result.

### Implications

The findings of this scoping review have implications for both research and practice. First, identifying the key components of the included approaches provides a basis for future research to explore particular components in depth, or to apply the approach in developing interventions. Moreover, identifying these key components provides an outline of potential techniques for practitioners to draw on should they wish to use a solution-focused approach with individuals with SITBs. This will contribute to the possibility for more effective care for individuals with SITBs. Additionally, demonstrating the use of these approaches with individuals with SITBs in a range of settings encourages confidence in implementing these approaches in practice. Outlining the various outcomes measured in several research studies enables future research to explore the same outcomes, allowing for future comparisons and synthesis of findings to strengthen the approach’s evidence base.

## Conclusions

This review is the first to provide a comprehensive overview of how solution-focused approaches are being used in treating SITBs. Overall, the approach is being delivered similarly to how it is delivered in other populations. We found a range of different components, modes of delivery, and delivery settings suggesting these approaches are used flexibly, which fits with the solution-focused approach’s ethos of being generic and adaptable. However, we did find specific adaptations of some components and the inclusion of several unique components directly relevant to treating SITBs. The lack of outcome data highlights the need for further evidence of solution-focused approaches being used with individuals with SITBs to provide more concrete evidence for their use. However, the included publications demonstrate the suitability and promise of these approaches for treating SITBs.

## Electronic supplementary material

Below is the link to the electronic supplementary material.

**Additional File 1**. Additional File 1_List of key components_revised.docx. List of key components. Table providing the full list of components identified in our review, with a summary and example for each.

**Additional File 2**. Additional File 2_List of outcome studies_final.docx. List of studies reporting outcomes. Table providing a list of the studies reporting outcome data, with each outcome and its raw data.

## Data Availability

Data sharing is not applicable to this article as no datasets were generated or analysed during the current study.

## Abbreviations

BDI: Beck Depression Inventory
CBT: Cognitive Behavioural Therapy
DBT-A: Dialectical Behavioural Therapy for children and young people
JBI: Joanna Briggs Institute
NICE: The National Institute for Health and Care Excellence
PCC: Population, Concept and Context
PRISMA-ScR: Preferred Reporting Items for Systematic Reviews and Meta-Analysis Extension for Scoping Reviews
SFBT: Solution-Focused Brief Therapy
SITB: Self-injurious thoughts and/or behaviour
