# Supplementary material for: Solution-focused approaches for treating self-injurious thoughts and behaviours: a scoping review
Source: BMC Psychiatry. 2024 Oct 1;24:646. doi: 10.1186/s12888-024-06101-7 (PMC11443805; doi:10.1186/s12888-024-06101-7)
Supplement: Supplementary file 1 — Additional File 1. Additional File 1_List of key components_revised.docx. List of key components. Table providing the full list of components identified in our review, with a summary and example for each. [file 12888_2024_6101_MOESM1_ESM.docx]

Additional File 1. List of key components. Table providing the full list of components identified in our review, with a summary and example for each. Components were classed as adapted for SITBs when SITBs were specifically addressed within the component description or examples given.

| Name | Adapted for SITBs | Summary | Example | References |
| --- | --- | --- | --- | --- |
| Acknowledge feelings | Yes | It is important to acknowledge client’s pain and suffering so the client feels understood, before moving on to building solutions and creating a possibility for change. | “Acknowledge - "from what you've told me about your situation, you have given me a pretty good idea about how difficult it is for you right now"” (1) | (1-5) |
| Activities within sessions | No | Sometimes tasks were described that could be performed within a session, to provide a basis for techniques the client can then access outside of therapy. | “5-minute mindfulness exercise. just breathing. Example: There is neither a right nor a wrong way to be mindful. Simply be who you directly experience yourself to be in the moment. If thoughts or emotions show up then observe them but do not believe or disbelieve them. As you practice, allow yourself to become more and more mindful of the sensations, thoughts, and feelings that are happening for you. Follow your breath. Simply watch your breath come in and go out of your body. This happens naturally. Feel the breath come in, feel the breath go out. Allow it to happen without getting in the way. If you want to, you can count your breaths, from one to ten. Once you have reached ten, go back to one. Just keep watching your breath. All kinds of content will come up when you sit. Your anger, depression, anxiety, low self-esteem – all these may surface. Just watch them come in and go out. As they appear, treat them with kindness, the way you would pat a visiting child on the head in acknowledgement of his presence.” (6) | (6, 7) |
| Assess suicide status | Yes | Some publications described using a specific risk assessment to assess suicide status either at intake or regularly. Most described asking direct questions to ask about suicidal thoughts and any plans/preparations, including using scales to assess current risk. Other publications reported suicidality being assessed more indirectly, from the client’s responses and descriptions in the session that can then be picked up on by the therapist if needed. | “2. Has it been so bad that you have thought about suicide? 3. How far have you gone with those thoughts? 4. How have you kept from acting on those plans?” (4) | (1, 3, 4, 8-17) |
| Beginning a session | No | One publication described the first 10 minutes as key for establishing rapport, empathy, hope and optimism. | “First 10 minutes: important to establish rapport and empathy. Hope and optimism should be communicated to and engendered.” (3) | (3) |
| Behavioural contract | Yes | One publication described the use of behavioural contracts. | “first session: began with establishing a specific behavioural contract which was agreed upon and duly signed the therapist and the client addressing the suicidal behaviour and the emergency drill. Further, the client was educated about depression and psychotherapy in general, and solution focused approach in specific emphasising the importance of working with a goal, taking responsibility for change and eliciting her expectations about her best hopes from therapy.” (14) | (14) |
| Bridging statements to tasks | No | Bridging statements were statements providing a rationale and context for the ‘homework task’ that was then to be set for the client. | “The bridge is simply a rationale for the homework suggestion: a statement or perhaps away of describing the suggestion that makes sense to the client. Often taking care to include the client’s language in describing the suggestion is sufficient as a bridge. often begin with such phrases as “I agree with you that . . . ” or “Because x [e.g., staying calm, taking care of your children] is so important to you right now . . .”” (4) | (1, 3, 4) |
| Circular questions | No | To help separate clients from their problems, help them to become aware of how interconnected they are and resources around them. | “allow members to become aware of how interconnected they are through the feelings and actions of other members. It may become clear, however, after discussing with clients their best hopes, and their resources, that utilizing a resource around them, a friend or local clinic, may be necessary. can help separate themselves from the problem they are experiencing, “If your cat were to say you are doing everything you need to do to make sure you are safe, what would you be doing?”” (12) | (12) |
| Collaborate on decisions | No | Emphasises working alongside the client and encouraging mutual participation in treatment. | “Mutual participation in treatment decisions—an aspect of what I would call collaborative treatment— has been associated with better outcome in a variety of health and mental health conditions. part of the therapeutic conversation would be a search for goals that are important, salient, and relevant to the client and on which we could cooperate.” (4) | (4, 13) |
| Compliments | Yes | Essential to highlight, reinforce, and orient clients to their strengths and skills. Should be based in reality and can be either direct/explicit or indirect. | “Should be based in reality and not given just to be nice or kind. Can be direct - a positive reaction or response to something the student has shared e.g. Wow I'm sure that must have been difficult for you to confront your friend and yet you found the courage to do it, or indirect - invite the student through a question to describe what they did and what worked well e.g. Wow how did you manage to find the courage to confront your friend?” (1) | (1, 3, 4, 6, 7, 10, 14, 18) |
| Constructive language | Yes | Intentional use of language to communicate therapist’s beliefs and hope for the client. | “Building toward co-constructive language, suggesting the client is “struggling with suicide” rather than are suicidal communicates that the clinician has hope about their future, believe in the capacity within themselves for change, and strengthens the therapeutic relationship” (12) | (12) |
| Containing crisis | Yes | Recognising not everything can be achieved but what would one thing you could do be that could make a difference. | “A focus on doing what can be done—rather than on doing everything, or doing the perfect thing—allows us to move forward. Doing what we can fits well with an emphasis on concrete, attainable, measurable goals, and, it specifically includes “harm reduction” approaches, for example, limiting or complicating access to an individual’s chosen method for suicide. "What is one call I could make?"” (4) | (4) |
| Coping questions | Yes | With clients who are particularly pessimistic or not ready to begin thinking of solutions, coping questions may be most appropriate. These highlights small, moment-to-moment achievements that are respectful of their position and focus on their capacity for survival. Includes inviting clients to consider how they are managing. | “coping questions: often more valuable in crisis. When clients begin to identify small successes in coping with the situation, questions about their goal and next steps may follow. Invite clients to think about coping in crisis situations by asking the following questions: “How did I manage to get out of bed this morning?”” (19) | (1-4, 9, 10, 16, 18, 19) |
| Create actions | No | A final step, encouraging clients to create actions for moving forward. | “The fourth stage of the GROW model is the wrap-up (W), which encourages clients to create specific action steps in order to move forward” (17) | (17) |
| Create positive expectancies | Yes | Use of presuppositional and positive language to convey hopefulness and therapist expectations that will give grounds for the client to feel similarly. | “Presuppositional questions in the first interview: ‘How are you going to sort this mess out?’, and, ‘What will be the first small step away from all this hopelessness and despair?’ Both these communicate worker hope and give grounds for client hope.” (3) | (3-6) |
| Describe and understand the current situation | Yes | Give appropriate attention to gaining an understanding of the client’s situation and clarify issues that relate to the clients presenting problem. | “important to invite the student to share His or her story about how he or she discovered it, what specifically it does for the student, when and where is it most likely to occur, and what effect this behavior has on the significant others in his or her life.” (8) | (1, 3, 7, 8, 10, 13, 15-18, 20) |
| Develop a working relationship with the client | No | Establish good rapport from the beginning to foster a cooperative therapeutic relationship. | “Start the process: establishment of good rapport. It is important to hear, acknowledge and validate the person’s stories before any interventions are undertaken.” (18) | (1, 7, 8, 18) |
| Education | Yes | Emphasised education including the mechanism of self-injury and building healthy ways of coping. | “the distribution of brochures to provide information that affirmed self-harm as a coping strategy but which also aimed to move the client from lack of awareness towards feeling motivated to secure community supports and enact change.” | (13, 15, 21) |
| Empowering statements | No | Encourage the client to move away from relying on the therapist. | “"Although these sessions are very important, what is even more important is what you think about, notice or put into practice between sessions. It involves hard work on your part. How willing are you to do this?". These are especially appropriate when practitioners sense that a client might be relying on the practitioner to ‘come up with the answers’ or ‘do something for them’.” (3) | (3) |
| Ending therapy | Yes | Both of a session and of the whole process. Wrapping up is important to highlight their skills and tasks they plan to complete. An assessment should be made for their discharge and any further support. | “make a point of wrapping up. Highlight coping skills and ability to deal with challenging times in the past. Confirm possible therapeutic tasks they plan to complete.” (1) | (1, 3, 4, 7, 9, 12, 17) |
| Evaluate progress | No | Evaluate the therapeutic process itself as well as any gains that may have been made. | “Provide a general evaluation of the consultation process. Evaluate the gains from the consultation process.” (7) | (6, 7, 14) |
| Explain the therapy process and approach | No | A brief introduction of how the service works and how the therapeutic approach works. | “A brief introduction to how the service works is all that is needed.” (3) | (3, 4, 6, 7, 13-15, 18) |
| Explore setbacks | No | Explore client’s achievements when encountering setbacks. | “What might be a sign that you are getting back on track again? What has surprised you about yourself? In situations where clients have encountered setbacks, it is especially important to acknowledge their achievements.” (20) | (20) |
| Family or social network involvement | Yes | Bringing in peers or family members can be beneficial in the client consents. | “Encouraging participation from family members can also be of valuable assistance when developing goals as well as other interventions for suicidal clients.” (16) | (4, 8, 12, 13, 16, 17, 22) |
| Feedback | No | Provide compliments and therapist observations in feedback at the end of the session or in feedback to take away with them as a reminder. | “Feedback: try to include a feedback component even in very brief helping conversations that may occur in relatively casual contexts. First and most important part of a solution-focused feedback message is compliments.” (4) | (4, 20) |
| Future focus questions | Yes | Encourages visualisation of a future scenario, so it becomes increasingly alive. Mainly using the miracle question but also other techniques that require imagining the future. | ““Suppose a miracle happens tonight, and the miracle is that you can cope with this situation, but you are unaware that the miracle has happened because you were asleep. What will you notice first thing tomorrow morning that shows you that the miracle has happened? What else will be different? When the miracle occurs, what will take the place of your pain and your thoughts of killing yourself?”” (19) | (1-7, 9-12, 14, 18-20, 23) |
| Homework | Yes | A variety of passive and active tasks for the client to try in between sessions. Ranging from thinking about, noticing, or doing something. Dependent on the capabilities of the client, which most deem a ‘noticing’ task as most appropriate for individuals who are suicidal. | “Solution-Enhancement Experiment: to keep track on a daily basis of useful self-talk and coping strategies employed to avoid the urge or temptation to cut, burn, or engage in any other form of self-harming behaviour. Once we identify with the student which specific self-talk tapes or coping strategies help the most, we want to have him or her increase these solution-building patterns of thinking and doing” (22) | (1, 3, 4, 6-8, 10, 18, 19, 22) |
| Identify and maintain change | No | Draw attention to any positive changes that have occurred, reinforcing the expectation of change and consolidating them. | “At the beginning of second and later sessions, positive change can be elicited by asking: – What has been better since we last met? – What has been different? At any point, positive changes or details of goals and solutions can be amplified and expanded by asking 'and what else?'” (10) | (4, 7, 10, 14, 20, 22) |
| Identify and plan for barriers | No | Slips are inevitable, barriers they are or may experience should be explored and different options for overcoming them evaluated. | “Self-harming students will experience inevitable slips throughout the course of intervention. Therefore, it is imperative that we prepare our students for how to constructively manage slips so that they do not escalate into prolonged relapsing and demoralizing crisis situations….• "What would you have to do to go backward at this point?" • "What did you learn from that slip, on Tuesday that you, will put to good use the next time you are faced with a similar stressful situation?" • "How were you able to stay on track on Monday?" "Wednesday?" ' ; • "Let's say we got together in 3 weeks" and you come in and tell me that you had a perfect vacation from counselling, what will you tell me you did to stay on track?" Finally, we need to address any student concerns or intervene as early as possible if he or she reports that the goal maintenance situation is beginning to unravel. Otherwise, the student will feel like he or she has returned back to square one.” (8) | (3, 7, 8, 14, 17, 22) |
| Identify and work on goals | Yes | When the client is ready, explore concrete goals that are small, workable and motivated by the client. Described as what they want rather than don’t want, and usually through asking what their treatment expectations are. | “It is important to find out what the patient wants. The therapist encourages the patient to start the session with an end point in mind. For example, the therapist may ask: ‘What would you like to happen as a result of this session?’” (18) | (1, 3, 4, 6-10, 12, 14, 16-18, 20, 22-24) |
| Identify exceptions | Yes | Usually exploring times when either part of their ‘miracle’ scenario has happened in the past, or times when they felt less suicidal. These are then elaborated upon helps to construct solutions in the present. | “Identify exceptions (times when the problem was absent, less frequent or less severe) after the miracle question. "I'm curious to know if a small part of your miracle has happened, or if a small part of it is happening today. Tell me more about the last time you felt a little better" "I'm curious about the last time you felt a little less suicidal. What were you doing or thinking differently than you are today?". Follow-up could include "How did you make that happen?...What else?...and what else?" "How did you decide to do that?" "What did you discover by doing that?" "What would happen if you tried that again?".” (1) | (1, 3, 4, 6-12, 16, 18, 21, 23, 24) |
| Identify strengths, skills, resources | Yes | Ask questions to elicit client strengths and resources that can then be highlighted explicitly in feedback and compliments. Encourage the client to use these in other contexts. | “Details were elicited on how she managed to keep up the change and what she did differently that contributed to this change in positive direction.” (14) | (1-4, 6, 8, 10-15, 17, 20-22) |
| Listen, select, build | No | Specific steps of listening for differences, selecting them and building upon them with the client. | “Listen, select, build: a fluid structure of conducting therapy with SFBT. It guides the clinician through assessment, while listening to moments of difference, selecting them, and then building collaboratively with the client their preferred future. refers to the intentional focus on the client’s language, their preferred futures, and exceptions to the problem, while avoiding assumptions about what the clients may be sharing”(12) | (12) |
| Motivation questions | Yes | Explore client motivations for further change. | “motivate further change - Motivation questions. Example: “How have you benefited now that you harm yourself less?” “What positive effects has this'.” (6) | (6) |
| Normalise feelings | Yes | Normalising suicidal feelings can be affirming and open up discussion of other options. | “Information giving: Common for suicidal clients to 'put on blinkers' regarding their options for the future. Normalise this. It is a relief for the suicidal caller to know that this is a common phenomenon within most people when they feel suicidal. Another piece of really useful information is this: ‘Suicidal feelings are a normal response by normal people to an abnormal set of circumstances.’” (3) | (1, 3) |
| Other people’s perspective questions | Yes | Exploring other people’s hopes for the client and what they would notice. Brings the work into the client’s social context and provides alternative viewpoints, as well as useful information on the client’s relationship resources. | “"What would other people notice you doing?". Relationship questions encourage students to view things from a third person perspective, which might be easier for them to provide details and ideas that can be used to develop goals.” (1) | (1-4, 9, 10, 18-20, 23) |
| Peer workers coach participants in the GROW model | Yes | Specific to the GROW model of coaching. | “Peer workers, 3 times per week for 4 weeks, coach participants through the steps in their aftercare plan using the framework of the GROW coaching model. Peer workers are trained in the SafeSide model of risk formulation and will escalate to the mental health clinician should they be concerned about a participant’s level of risk.” (17) | (17) |
| Post-treatment evaluation and referrals | No | Referrals post-treatment to other teams for evaluation and follow-up. | “post-treatment evaluation: the client was referred to the team of Psychiatrist and Clinical Psychologist who assessed her” (14) | (14) |
| Pre-session change | No | Explore what may have happened between asking for help and the session, recognising they are doing something different by coming to the session. | “Pre-session change: typically asked about early in the first session: What has been different since you made the decision/booked the appointment to come here? Recognition the client is already 'doing something different' by asking for help.” (10) | (3, 4, 10, 12) |
| Problem-free talk | No | Can interrupt problem patterns of thinking and help to build rapport by engaging with the client rather than the problem. | “an adapted form of problem free talk runs as follows: ‘Before things got as they have been, in what sorts of ways did you spend your free time?’ or, ‘Before you got as low as you did, what sorts of things did you do in the day that interested you?’.” (3) | (1, 3, 12) |
| Rating and scaling questions | Yes | Usually on a 1 to 10 scale, used to rate a number of different things. Allows the client to reflect and communicate their current state, as well as give the therapist valuable information about their status and progress. Help to create small manageable steps. | “"on a scale of 1 to 10, with 1 not being well at all and 10 being very well, how well do you feel right now as you're talking with me?". If they are not doing well, "on a scale of 1 to 10, with 1 being close and 10 being not close at all, how close do you feel right now to ending your life?" "on a scale of 1 to 10, with 1 being very suicidal and 10 being not suicidal at all, how suicidal do you feel right now?".” (1) | (1-3, 6, 7, 9-12, 14, 16, 18-20, 23, 24) |
| Recovery plan questions | Yes | When prevention plans fail, a recovery plan can be mapped, usually derived from how they recovered from a previous crisis. | “When prevention plans fail or are not put into practice, a recovery plan may be mapped out, especially with clients who have severe mental problems like psychosis, major depressive episodes, or suicidal thoughts. This can usually be derived from inviting clients to think about what happened as they regained equilibrium after a previous crisis or hospitalization: “What was I doing when I started to feel better again?” “What usually happens when I begin to emerge from one of my depressive episodes?” “What did I learn from previous crises/hospitalizations that may be helpful in this situation?”” (19) | (19) |
| Reminders | Yes | Concrete reminders of the client’s strengths, hopes, accomplishments that can be needed during difficult times. | “Concrete reminders to clients of hopeful signs, reasons for living, positive plans, and relationship supports are useful and necessary when clients are experiencing after-effects of trauma or people who are in suicidal crisis. Reminders may include notes or lists (e.g., of reasons to live), tattoos or symbolic objects. “Rainy day letters” are written when clients are feeling well and hopeful, including clients' strengths, accomplishments, reasons for living and future hopes, and may provide much needed reminders during difficult times.”(2) | (2) |
| Resource form | Yes | Identification of emergency contacts and services for the therapist and client. How it will be used is agreed giving a clear plan of action if in crisis. | “Providing the client with the opportunity to identify resources in their community allows them to see their own strength in creating an action plan for support. As the therapist asks questions such as “Who would you need to reach out to in order to receive the care and support you need?” or “Who in your life have you reached out to in the past that was helpful in overcoming trials or challenges?” (12) | (12) |
| Restate negatives in positive terms | No | Rather than clients stating what they don’t want, or what they want less of, ask them to rephrase as what they do want instead. | “Another common type of response to the miracle question is that something negative will be absent–e.g., “My parents won‘t be yelling at me”, “I won’t feel so hopeless”. This kind of response can be restated in positive terms that allow for positive goal formulation and exception-finding by asking: – what will be happening instead of [the negative event]?” (10) | (10) |
| Safety planning | Yes | Identify what safety looks like for the client and what steps could be taken to keep them safe. | “27. What else needs to happen between now and when we meet again to help you keep living? 28. Who in your life will want to help with this plan? How can that person make a difference? Shall I call or do you want to make the call?” (4) | (4, 16) |
| Solution talk | Yes | Problem definition is important, then move on to understanding what can be a solution for the client. | “Careful listening and clinical judgement help to determine the most appropriate timing for introducing "solution-talk". Understanding how the individual views suicide as a personal solution is a first step toward understanding what else could serve as a solution for this person.” (10) | (10) |
| Stance | No | Important to take a ‘solution-focused stance’ as a therapist. | “More important than any question or technique is the solution-focused stance: respectful, curious, mindful.” (23) | (23) |
| Think break | No | Therapist reflects on the session and prepares feedback. | “A core component of the new assessment was the ‘think break’. As the assessment came to a close the therapist asked the patient’s permission to take a few minutes to gather his or her thoughts – in reality the thinking is done alongside the doing. The therapist reflected on what he or she had learned about the person during that session and prepared for the feedback.” (18) | (18) |
| Use client’s language | No | Use client language and beliefs to help foster a cooperative relationship. | “use the clients’ strengths, key words, beliefs, and metaphors connected to their major skill areas as much as possible” (22) | (22) |
| Use of themes in art therapy groups | No | Themes may be appropriate to suggest in art therapy groups. | “There are varying opinions regarding the use of a set theme in an art therapy group (Liebmann, 2012, p. 368). Liebmann (2012) highlights the circumstances in which a theme may be appropriate, explaining that “the choice of approach may depend on the client group, the purpose of the group, the time available, and the preferred style of the therapist” (p. 370). A theme may benefit group process by increasing group cohesion, and it is especially appropriate for short-term treatment as a theme may help members focus on goals (pp. 369-370). Themes discussed the use of the theme 'self-care.'” (21) | (21) |
| Validate feelings | Yes | Validate the client’s feelings. | “Validate - "based on everything you've shared with me, it's understandable that you are having some suicidal thoughts".” (1) | (1) |

1. Buchholz Holland C. SFBT in Action: Mental Health and Suicidal Ideation. In: Kim J, editor. Solution-Focused Brief Therapy in Schools: A 360-Degree View of the Research and Practice Principles. New York: Oxford Academic; 2017. p. 126-52.

2. Fiske H. Preventing suicide in the aftermath of trauma. In: Froerer A, editor. Solution-focused brief therapy with clients managing trauma. New York: Oxford Academic; 2018. p. 64-83.

3. Henden J. Preventing suicide: The solution focused approach. New York, NY: John Wiley & Sons Ltd; 2017 2008.

4. Fiske H. Hope in action: Solution-focused conversations about suicide. New York, NY: Routledge/Taylor & Francis Group; 2008 2008.

5. Cole-King A, O'Neill S. Suicide Prevention: Identification, Intervention and Mitigation of Risk. In: Gask L, Kendrick T, Peveler R, editors. Primary Care Mental Health. Cambridge; New York, NY: Cambridge University Press; 2018. p. 103-24.

6. Tapolaa V, Lappalainen R, Wahlstrom J. Brief intervention for deliberate self harm: An exploratory study. Suicidology Online. 2010;1:95-108.

7. Ayar D, Sabanciogullari S. The effect of a solution‐oriented approach in depressive patients on social functioning levels and suicide probability. Perspectives in Psychiatric Care. 2020;57(1).

8. Selekman M. Integrative, solution-oriented approaches with self-harming adolescents. In: Franklin C, Harris MB, Allen-Meares P, editors. The School Practitioner’s Concise Companion to Health and Well Being. New York: Oxford University Press; 2008. p. 109-18.

9. Kondrat D, Teater B. Solution-focused therapy in an Emergency Room setting: Increasing hope in persons presenting with suicidal ideation. Journal of Social Work. 2012;12(1):3-15.

10. Fiske H. Applications of Solution-focused Brief Therapy in Suicide Prevention. In: De Leo D, Schmidtke A, Diekstra RFW, editors. Suicide Prevention: A Holistic Approach: Kluwer Academic; 1998. p. 185-97.

11. Fiske H, editor 20 GOOD REASONS TO USE SFBT IN SUICIDE PREVENTION. Solution-Focused Brief Therapy Association Conference; 2017; Santa Fe, NM.

12. Finlayson B, Jones E, Pickens J. Solution focused brief therapy telemental health suicide intervention. Contemporary Family Therapy. 2023;45:49-60.

13. McAllister M, Moyle W, Billett S, Zimmer-Gembeck M. 'I can actually talk to them now': Qualitative results of an educational intervention for emergency nurses caring for clients who self‐injure. Journal of clinical nursing. 2009;18(20):2838-45.

14. Baijesh A, Suresh Kumar P. Solution Focused Brief Therapy (SFBT) In the Treatment of Depression and Suicidal Ideation: A Case Study. Case Studies Journal ISSN (2305-509X). 2018;7(1):61-5.

15. McAllister M, Zimmer-Gembeck M, Moyle W, Billett S. Working effectively with clients who self-injure using a solution focused approach. International Emergency Nursing. 2008;16(4):272-9.

16. Guterman J. Suicide. In: Guterman J, editor. Mastering the art of solution-focused counseling. 2nd ed: American Counseling Association; 2013. p. 165-75.

17. Bliokas V, Hains A, Allan J, Lago L, Sng R. Community-based aftercare following an emergency department presentation for attempted suicide or high risk for suicide: study protocol for a non-randomised controlled trial. BMC Public Health. 2019;19.

18. Wiseman S. Brief intervention: reducing the repetition of deliberate self-harm. Nursing Times. 2003;99(35):34-6.

19. Bannink F. Crisis and Suicide. In: Bannink F, editor. 101 Solution-focused Questions for Help with Depression: W. W. Norton & Company; 2015.

20. Laydon C, Mackenzie S, Jones S, Wilson-Stonestreet K. Solution-focused therapy for clients who self-harm. Nursing Times. 2008 2023-2-10.

21. Lefrançois-Crotty J. Self-Care Through Art Therapy: A Group Intervention Design for Female Adolescents who Self-Harm: Concordia University; 2013.

22. Selekman M. Integrative, Solution-Oriented Approaches With Self-Harming Adolescents. In: Franklin C, Harris MB, Allen-Meares P, editors. The school services sourcebook : A guide for school-based professionals. New York, NY: Oxford University Press; 2006. p. 321-8.

23. Fiske H. Solution-focused brief therapy and suicide prevention. International Journal of Brief Therapy and Family Science. 2017;7(1):1-2.

24. Rhee W, Merbaum M, Strube M, Self S. Efficacy of brief telephone psychotherapy with callers to a suicide hotline. Suicide and Life-Threatening Behavior. 2005;35(3):317-28.
