# Supplementary material for: Solution-focused approaches for treating self-injurious thoughts and behaviours: a scoping review
Source: BMC Psychiatry. 2024 Oct 1;24:646. doi: 10.1186/s12888-024-06101-7 (PMC11443805; doi:10.1186/s12888-024-06101-7)
Supplement: Supplementary file 2 — Additional File 2. Additional File 2_List of outcome studies_final.docx. List of studies reporting outcomes. Table providing a list of the studies reporting outcome data, with each outcome and its raw data. [file 12888_2024_6101_MOESM2_ESM.docx]

Additional File 2. List of outcomes from publications that measured outcomes and their raw scores.

Table 1. Raw scores from quantitative outcome measures, reported for participants who received a solution-focused intervention

| Outcome measured | K | N | Baseline | Follow-up |
| --- | --- | --- | --- | --- |
| BDI | 4^1-4^ | 1^1^ | 14 | 4^a^ |
|  |  | 85^2^ | 30.85 (3.05) | 16.92 (2.87)^b^ |
|  |  | 62^3^ | 36.32 (4.23) | - |
|  |  | 16^4^ | 31.33 (9.93) | 25.00 (13.57)^c^ |
| Beck VAS | 1^2^ | 85 | 72.69 (6.52) | 47.69 (7.57)^b^ |
| BSI | 1^2^ | 85 |  |  |
| Overall |  |  | 84.69 (10.79) | 42.69 (9.13)^b^ |
| Psychoticism |  |  | 9.46 (1.00) | 4.62 (1.11)^b^ |
| Paranoia |  |  | 9.77 (1.22) | 4.92 (1.17)^b^ |
| Phobias |  |  | 5.31 (1.17) | 2.54 (1.03)^b^ |
| Hostility |  |  | 6.15 (1.38) | 2.92 (0.70)^b^ |
| Anxiety |  |  | 8.54 (1.34) | 2.62 (0.84)^b^ |
| Depression |  |  | 14.62 (1.91) | 9.31 (2.00)^b^ |
| Interpersonal sensitivity |  |  | 9.38 (1.16) | 4.92 (1.30)^b^ |
| Obsession-compulsion |  |  | 10.08 (1.77) | 5.54 (1.14)^b^ |
| Somatisation |  |  | 5.54 (1.56) | 1.62 (0.66)^b^ |
| Miscellaneous items |  |  | 7.00 (1.10) | 4.08 (1.00)^b^ |
| Satisfaction with Life scale | 1^2^ | 85 | 10.54 (1.42) | 15.08 (1.77)^b^ |
| Deliberate self-harm | 1^4^ | 16 | 2.29 (1.25) | 0.43 (0.54)^a^ |
| 15D – Health Quality | 1^4^ | 16 | 28.67 (4.97) | 24.67 (5.85)^c^ |
| Difficulties in emotion regulation scale DERS | 1^4^ | 16 |  |  |
| Overall |  |  | 104.00 (20.56) | 90.00 (18.49)^c^ |
| Emotion non-acceptance |  |  | 21.00 (4.78) | 18.00 (5.51)^c^ |
| Impulse dyscontrol |  |  | 15.83 (4.83) | 12.67 (3.88)^c^ |
| Goal-directed difficulties |  |  | 15.00 (3.85) | 12.67 (3.67)^c^ |
| Emotion non-awareness |  |  | 13.33 (3.88) | 13.17 (3.55)^c^ |
| Lack of ER strategies |  |  | 23.67 (4.03) | 22.33 (4.80)^c^ |
| Lack of clarity |  |  | 15.17 (3.77) | 11.17 (2.04)^c^ |
| Action and Acceptance Questionnaire (AAQ) | 1^4^ | 16 | 31.17 (12.73) | 36.00 (12.33)^c^ |
| Beck Anxiety Inventory BAI | 1^4^ | 16 | 23.00 (13.07) | 20.00 (14.33)^c^ |
| Social Adaptation Self-Evaluation Scale | 1^3^ | 62 | 28.32 (10.21) | 33.74 (6.19)^d^ |
| Suicide Probability Scale | 1^3^ | 62 |  |  |
| Overall |  |  | 77.00 (13.84) | 63.32 (14.80)^d^ |
| Negative Self and Exhaustion |  |  | 41.42 (8.87) | 32.90 (10.14)^d^ |
| Dissociation From Devotion to Life |  |  | 18.32 (8.80) | 15.42 (6.56)^d^ |
| Anger |  |  | 18.87 (6.43) | 14.61 (6.20)^d^ |
| Suicidal Ideation Scale | 1^1^ | 1 | 107 | 27^a^ |
| Beck Hopelessness Scale | 1^1^ | 1 | 13 | 1^a^ |
| Professional self-concept scale | 1^5^ | 71 | 3.24 (0.27) | 3.23 (0.28)^b^ |
| Perceptions of nursing scale | 1^5^ | 71 |  |  |
| Belief in distinctive role of nursing |  |  | 3.67 (0.64) | 3.86 (0.64)^b^ |
| Nursing is problem oriented |  |  | 3.72 (0.43) | 3.76 (0.29)^b^ |
| Nursing is strengths oriented |  |  | 3.62 (0.50) | 3.88 (0.50)^b^ |
| Satisfied with skills |  |  | 3.28 (0.78) | 3.67 (0.67)^b^ |
| In need of more skills |  |  | 4.04 (0.72) | 4.07 (0.58)^b^ |
| Believe in social role |  |  | 3.56 (0.85) | 3.85 (0.78)^b^ |
| Clear purpose |  |  | 3.50 (0.42) | 3.50 (0.49)^b^ |
| Client’s problem/dx takes priority |  |  | 3.30 (0.91) | 3.15 (0.97)^b^ |
| Working with clients’ strengths not reality |  |  | 3.48 (0.80) | 3.44 (0.93)^b^ |
| Skills in being proactive |  |  | 3.56 (0.75) | 3.74 (0.76)^b^ |
| Value being person-centred |  |  | 3.96 (0.76) | 3.70 (0.95)^b^ |
| Nursing’s identity hard to explain |  |  | 3.22 (1.09) | 3.00 (1.04)^b^ |
| More focus at social level |  |  | 3.52 (0.75) | 3.19 (0.83)^b^ |
| Develop better technical skills |  |  | 3.74 (0.90) | 3.89 (0.85)^b^ |
| Learn more about solution focused |  |  | 3.67 (0.73) | 3.70 (0.67)^b^ |
| Post-session change | 1^6^ | 40 | Identified by 78% | |
| Patient satisfaction | 1^6^ | 40 | 96% felt the service was more than satisfactory | |

^a^6 months, ^b^post-treatment/intervention, ^c^4 months, ^d^3 months

K = number of studies

N = number of participants overall in the study

Table 2. Qualitative outcomes

| Data/Study type | Author | Findings | |
| --- | --- | --- | --- |
| Staff and participant anecdotal reports | Laydon et al. ^7^ | *“Feedback from staff is encouraging”* | |
|  |  | *“Anecdotal evidence of the potential benefit of follow-up can be seen in the following letter from one of our assessments: 'Your letter arrived this morning. The first time I tried to read it I couldn't for tears. I went back two hours later and read it 10 times'. 'I will keep it with me and read it regularly I'm sure'. 'You have no idea what you have done for me! You were a complete stranger that I was dreading meeting...the 'miracle' is the future’.”* | |
| Participant anecdotal report (by clinician) | Baijesh and Kumar ^1^ | *“client responded well to the compliments given on the efforts and achievements and was pleased with her progress.”* | |
| Interviews with nurses 2 weeks after receiving training in the intervention – themes identified using content analysis | McAllister et al. ^8^ | Themes  Equipped with new  response skills  Perspective  transformation  Positive Effects  Lessons for the  future  Nil effect | Subthemes  Acknowledgement  Strategic assessment  Proactive responses  A framework for care  Referral skills and resources  Team approach  Transferable skills  Seeing the complexity of self-harm  Seeing links between blame and destruction  The little things can facilitate change  The value of using a person-focus  Triggering change, rather than providing cure  Seeing self-harm as a cycle  More confidence  Positive attitudinal shift  Efficacy  Resources are needed to ensure capacity to care  Provide the intervention to all clinical staff  Provide emotional support following the intervention  No perceived relevance to practice |

1. Baijesh A and Suresh Kumar P. Solution Focused Brief Therapy (SFBT) In the Treatment of Depression and Suicidal Ideation: A Case Study. *Case Studies Journal ISSN (2305-509X)* 2018; 7: 61-65.

2. Rhee W, Merbaum M, Strube M, et al. Efficacy of brief telephone psychotherapy with callers to a suicide hotline. *Suicide and Life-Threatening Behavior* 2005; 35: 317-328. DOI: doi:10.1521/suli.2005.35.3.317.

3. Ayar D and Sabanciogullari S. The effect of a solution‐oriented approach in depressive patients on social functioning levels and suicide probability. *Perspectives in Psychiatric Care* 2020; 57. DOI: doi:10.1111/ppc.12554.

4. Tapolaa V, Lappalainen R and Wahlstrom J. Brief intervention for deliberate self harm: An exploratory study. *Suicidology Online* 2010; 1: 95-108. DOI: doi:.

5. McAllister M, Zimmer-Gembeck M, Moyle W, et al. Working effectively with clients who self-injure using a solution focused approach. *International Emergency Nursing* 2008; 16: 272-279. DOI: doi:10.1016/j.ienj.2008.05.007.

6. Wiseman S. Brief intervention: reducing the repetition of deliberate self-harm. *Nursing Times* 2003; 99: 34-36. DOI: doi:.

7. Laydon C, Mackenzie S, Jones S, et al. Solution-focused therapy for clients who self-harm. *Nursing Times*. 2008.

8. McAllister M, Moyle W, Billett S, et al. 'I can actually talk to them now': Qualitative results of an educational intervention for emergency nurses caring for clients who self‐injure. *Journal of clinical nursing* 2009; 18: 2838-2845. DOI: doi:10.1111/j.1365-2702.2008.02540.x.
